# Supplementary material for: Prevalence of and Risk Factors for Iron Deficiency in Twin and Singleton Newborns
Source: Nutrients. 2022 Sep 17;14(18):3854. doi: 10.3390/nu14183854 (PMC9500937; doi:10.3390/nu14183854)
Supplement: Supplementary file 1 [file nutrients-14-03854-s001.zip › nutrients-1917684-supplementary.pdf]

Table S1. Immunoassay details.

Analyte	Immunoassay manufacturer	Catalogue number	Volume added to plate	Sample dilution	Minimal detectable conc.	Highest stand-ard conc.	Lowest Standard conc.	QC high conc. (ng/mL)	QC low conc. (ng/mL)
Hepcidin-25	DRG International, Springfield, NJ, USA	EIA5782R	20 uL	No dilution	0.153 ng/mL	81 ng/mL	1 ng/mL	34.3	4.71
Ferritin	DRG International, Springfield, NJ, USA	EIA4097R	25 uL	No dilution	NA	800 ng/mL	10 ng/mL	NA	NA
sTfR	DRG International, Springfield, NJ, USA	EIA5925R	100 uL	51	0.145 ng/mL	160 ng/mL	10 ng/mL	64.7	14.5
CRP	R&D Systems/Biotechne, Minneapolis, MN, USA	DCRP00	50 uL	10 *	0.01 ng/mL	50 ng/mL	0.78 ng/mL	NA	NA
IL-6	eBioscience/ThermoFisher, Waltham, MA, USA	88-7066-22	100 uL	2	2.0 pg/mL	200 pg/mL	3.13 pg/mL	NA	NA

* 4 samples were re-measured at 100× or 500× dilution after determined outliers at the initial 10× dilution. Conc., concentration; QC, quality control; NA, not available; sTfR, soluble transferrin receptor; CRP, c-reactive protein; IL-6, Interleukin 6.

.

Table S2. Maternal, pregnancy and neonatal characteristics associated with neonatal ID.

Characteristic	IS		ID		OR	95% CI
	n	%	*n*	%		
**Race**						
White	82	0.84	16	0.16		
Black	17	0.63	10	0.37	11.88	(0.92, 94.12)
Other	4	1.00	0	0.00	1.00	(1.00, 1.00)
Maternal age, years						
<25	31	0.79	8	0.21		
25–<35	54	0.78	15	0.22	0.81	(0.06, 4.54)
≥35	18	0.86	3	0.14	0.29	(0.01, 5.36)
Gravidity						
1	43	0.78	12	0.22		
2+	60	0.81	14	0.19	0.75	(0.11, 5.4)
Parity						
0	51	0.80	13	0.20		
1+	52	0.80	13	0.20	0.97	(0.14, 6.84)
BMI late pregnancy						
<30	14	0.74	5	0.26		
30–<35	12	0.75	4	0.25	0.75	(0.02, 33.16)
≥35	36	0.88	5	0.12	0.17	(0.01, 7.07)
Anemic (Hb <11 g/dL)						
No	41	0.89	5	0.11		
Yes	25	0.74	9	0.26	7.32	(0.52, 134.5)
Preeclampsia						
No	76	0.78	21	0.22		
Yes	28	0.80	7	0.20	0.86	(0.09, 7.81)
Twin status						
Singleton	19	0.79	5	0.21		
Twin	84	0.80	21	0.20	0.87	(0.11, 10.26)
Twin type						
Di-di	74	0.79	20	0.21		
Mono-di	11	0.79	3	0.21	0.99	(0.04, 27.12)
Gestational age (GA), weeks					1.35	(0.77, 1.81)
Preterm (GA <37 weeks)						
No	26	0.87	4	0.13		
Yes	77	0.78	22	0.22	2.38	(0.17, 15.67)
Delivery context						
Term delivery	26	0.84	5	0.16		
Spontaneous PTB	36	0.73	13	0.27	2.31	(0.19, 28.27)
Provider-initiated PTB	42	0.81	10	0.19	1.14	(0.09, 13.82)
Delivery type						
Vaginal	38	0.78	11	0.22		
C-section	65	0.81	15	0.19	0.51	(0.08, 3.94)
Birth weight (BW), g					1.00	(0.999, 1.0024)
LBW (BW <2500 g)						
No	42	0.81	10	0.19		
Yes	61	0.79	16	0.21	0.69	(0.13, 4.71)
VLBW (BW <1500 g)						
No	92	0.78	26	0.22		
Yes	11	1.00	0	0.00	1.00	(0.00, 5.66)
Sex						
Male	48	0.79	13	0.21		
Female	55	0.81	13	0.19	0.21	(0.07, 2.39)

ORs and 95% confidence intervals are from mixed effect models with random effects for twin pairs. Abbreviations: BMI, body mass index; BW, birth weight; CI, confidence interval; GA, gestational age; Hb, hemoglobin; ID, iron deficient; IS, iron sufficient; LBW, low birthweight; OR, odds ratio; PTB, preterm birth; VLBW, very low birthweight.

Table S3. Maternal, pregnancy and neonatal characteristics associated with continuous iron biomarkers in linear mixed effects regression models.

	SF		sTfR		Hepcidin	
	B	95% CI	B	95% CI	B	95% CI
Race						
White						
Black	−0.55	(−0.97, −0.13)	0.18	(0.0, 0.36)	−0.23	(−0.82, 0.35)
Other	0.08	(−1.01, 1.16)	−0.07	(−0.52, 0.39)	−0.50	(−1.99, 0.99)
Maternal age, years						
<25						
25–<35	0.12	(−0.3, 0.53)	−0.06	(−0.24, 0.11)	−0.19	(−0.74, 0.36)
≥35	0.13	(−0.42, 0.68)	−0.14	(−0.38, 0.09)	−0.42	(−1.16, 0.32)
Gravidity						
1						
2+	0.08	(−0.29, 0.45)	−0.05	(−0.21, 0.11)	−0.11	(−0.61, 0.39)
Parity						
0						
1+	−0.01	(−0.38, 0.35)	−0.04	(−0.19, 0.12)	−0.18	(−0.67, 0.31)
Late pregnancy BMI						
<30						
30–<35	0.02	(−0.65, 0.68)	−0.04	(−0.33, 0.25)	0.27	(−0.77, 1.31)
≥35	−0.13	(−0.68, 0.43)	0.10	(−0.14, 0.34)	−0.12	(−0.98, 0.74)
Anemic (Hb <11 g/dL)						
No						
Yes	−0.13	(−0.56, 0.3)	−0.12	(−0.31, 0.07)	−0.25	(−0.94, 0.43)
Preeclampsia						
No						
Yes	−0.10	(−0.51, 0.31)	0.15	(−0.02, 0.32)	−0.44	(−0.98, 0.11)
Twin status						
Singleton						
Twin	0.10	(−0.31, 0.5)	−0.28	(−0.44, −0.11)	0.04	(−0.52, 0.61)
Twin type						
Di-di						
Mono-di	−0.04	(−0.68, 0.59)	0.10	(−0.11, 0.32)	0.41	(−0.43, 1.25)
Gestational age, weeks	0.01	(−0.07, 0.09)	0.02	(−0.01, 0.05)	0.12	(0.02, 0.22)
Preterm (<37 weeks)						
No						
Yes	−0.47	(−0.89, −0.06)	−0.01	(−0.19, 0.17)	−0.67	(−1.23, −0.12)
Delivery type						
Vaginal						
C-section	0.11	(−0.22, 0.45)	−0.03	(−0.18, 0.12)	−0.36	(−0.83, 0.11)
Birth weight, g	−0.0001	(−0.0004, 0.0002)	0.0001	(−0.0001, 0.0002)	0.0002	(−0.0002, 0.0006)
LBW (<2500 g)						
No						
Yes	0.03	(−0.25, 0.32)	−0.09	(−0.23, 0.04)	−0.28	(−0.72, 0.17)
VLBW (<1500 g)						
No						
Yes	0.16	(−0.25, 0.57)	−0.01	(−0.22, 0.2)	−0.15	(−0.82, 0.52)
Sex						
Male						
Female	0.32	(0.11, 0.53)	−0.07	(−0.19, 0.04)	0.30	(−0.06, 0.67)

Coefficients and 95% confidence intervals are from mixed effect models with random effects for twin pairs. Confidence intervals (CI) that do not contain zero indicate consistency with a non-zero association between the variable and the cord blood iron biomarker (i.e., statistical significance). Abbreviations: BMI, body mass index; BW, birth weight; CI, confidence interval; GA, gestational age; Hb, hemoglobin; ID, iron deficient; IS, iron sufficient; LBW, low birthweight; OR, odds ratio; PTB, preterm birth; SF, serum ferritin; sTfR, soluble transferrin receptor; VLBW, very low birthweight.
